# Control of Cutaneous Leishmaniasis Using Geographic Information Systems from 2010 to 2014 in Khuzestan Province, Iran

**DOI:** 10.1371/journal.pone.0159546

**Published:** 2016-07-28

**Authors:** Mojdeh Ostad, Sadegh Shirian, Fatemeh Pishro, Tahereh Abbasi, Armin Ai, Farideh Azimi

**Affiliations:** 1 Department of Geography, Ahvaz Islamic Azad University, Ahvaz, Iran; 2 Department of Pathology, School of Veterinary Medicine, Shahrekord University, Shahrekord, Iran; 3 Brain and Spinal Cord Injury Research Center, Neuroscience Institute, Tehran University of Medical Science, Tehran, Iran; 4 Shiraz Molecular Pathology Research Center, Dr Daneshbod Lab, Shiraz, Iran; 5 Department of Water Resource Engineering, Shiraz Islamic Azad University, Shiraz, Iran; 6 Tehran University of Medical Sciences, Tehran, Iran; Academic Medical Centre, NETHERLANDS

## Abstract

**Background:**

Cutaneous leishmanisis (CL) is found worldwide and is considered to be endemic in 88 countries such as Iran. Geographic information system (GIS) is a method that can create, archive, analyze traditional map and place data of the disease distribution. The aim of this study was to produce distributional maps of CL over five years and evaluate the role of GIS in control of CL in Khuzestan province where an endemic area of CL in Iran is.

**Methods:**

CL epidemiological data on the District and village levels for the period 2010–2013 were provided as census by health surveillance system in all counties and in control diseases center (CDC) of Khuzestan province. After collection of CL data, the collected data of CL from 2010 to 2013 were analyzed using GIS. The collected data of CL from 2010 to 2013 was analyzed using GIS. The endemic areas of CL during 2010–2013 were recognized using GIS maps and the control programs of CL were done in these regions based on epidemiological situation and the stratification of risk areas.

**Results:**

During the study period, there were 4672 recorded cases of clinical cases of CL by Khuzestan Health Center. Data of GIS referring to CL patients showed that center and eastern districts of Khuzestan had a significant number of cases. In 2014 that control program was done, ten distinct of Khuzestan Province didn’t show any cases of the disease.

**Conclusion:**

In conclusion, analyses of data distributed in the geographic spaces are increasingly appreciated in leishmaniasis control management. GIS tools promoted greater efficiency in making decisions and planning activities in the control of vector born disease such as leishmaniasis.

## Introduction

Leishmaniasis is a worldwide vector-borne disease affecting 88 countries in almost every continent and presents three different forms visceral, cutaneous, and mucocutaneous [1, 2]. It is estimated that more than 350 million people are at risk, 15 million are already infected, and 1.5–2 million are infected annually [3, 4]). Despite advances in diagnosis and treatment of leishmaniasis, it is now considered as a severe public health problem particularly in developing countries and Iran and a great economic burden on the health resources [5, 6]. Various *Leishmania* species including members of the *Leishmania* subgenus (*L*. *infantum*, *L*. *major* and *L*. *tropica*) are prevalent in Iran [7]. Cutaneous leishmaniasis (CL) is a disabling form of the disease with remarkable variation in the clinical manifestations. CL lesions are multiple, frequently self-healing in the old world and is the most common form of the disease in Iran [2]. Khuzestan province in the south of Iran is one of the important foci of the disease. In the Iran, these diseases are transmitted by the bite of the infected female *Phlebotomus* sand flies. Among the various ecological factors associated to the distribution of particular *Phlebotomus* species in the Old World and *Lutzomyia* species in the New World, global climatic changes seems to be a critical factor [8, 9]. Leishmaniasis is particularly endemic in the southern and southwestern region of Iran in which the Kuzestan province is located [10, 11]. Many vector-bone diseases including Lyme disease, malaria, Fasciola, Rift Valley fever, and Schistosoma have a focal area, where the spatial distribution of the parasite, host, vector and required environmental conditions coincide and geographic information systems (GIS) are used for description of these diseases [12]. Leishmaniasis is another vector-borne disease related to environmental change [13]. Distribution and abundance of vectors and reservoirs of leishmaniasis is directly or indirectly affected by different factors including environmental factors.

This disease presents as various variation in its geographical areas of occurrence, with a focal distribution [14, 15]. Geographic information system is a method that can create, archive, analyse traditional map and place data of diseases distribution in epidemiology and combine these with environmental data [12]. A development of the GIS during the two last decades have provided more powerful and efficient tools to investigate spatial patterns and is worthwhile tool in studying infectious diseases [16]. To the best of our knowledge, the control of CL by using GIS has been not considered before. Our study attempts to evaluate the role of GIS in control of CL in Khuzestan province, Iran.

## Material and Methods

### Study area and population

Khuzestan province is located in the southwestern of Iran at 29°57′ -33°0′N, 47°38′-50°32′ E and contains approximately 3.9% of the total surface area of Iran including 63,238 km^2^. This province covers 126 counties; its climate is quite dusty and dry, with warm summers, mild winters and a great deal of sunshine throughout the year. The average temperature varies from 42.4°C in summer to 35.3°C, 25.3°C, and 13.1°C in autumn, spring, and winter, respectively. The study subjects consisted of all the residents in the counties of Khuzestan province, including 126 counties [17]. The province has an estimated population of 4700000 inhabitants.

This work aimed to report the experience of control of CL in Khuzestan Province, Southern Iran. Cutaneous leishmaniasis epidemiological data on the District and village levels for the period 2010–2013 were provided as census by health surveillance system in all counties and in control diseases center (CDC) of Khuzestan province. After collection of CL data, all the collected data was analyzed by using Arc GIS 9.3 software and spatial analysis. Cutaneous leishmaniasis incidence was evaluated by GIS maps with 1:25,000 scale. The study analyzed data of CL from 2010 to 2013 using GIS. The endemic areas of CL during 2010–2013 were recognized using GIS maps and the control programs of CL were done in these regions based on epidemiological situation and the stratification of risk area at the early months of 2014, especially one month before sandflies activity. Control measures of CL were done considering the following aspects: annual programming of activities to control the rodent reservoir and chemical control of sand fly vectors. Incidence and distribution of CL was obtained from CDC of Khuzestan Province in the end of 2014 and analyzed again using GIS.

## Results

Evaluation of the annuals control measures for CL in Khuzestan Province is reported here. During the study period, there were 4672 recorded cases of clinical cases of CL by Khuzestan Health Center. Out of 4672 patients, 2805 (60%) patients were male and 1867 (40%) were female.

**Fig 1:**
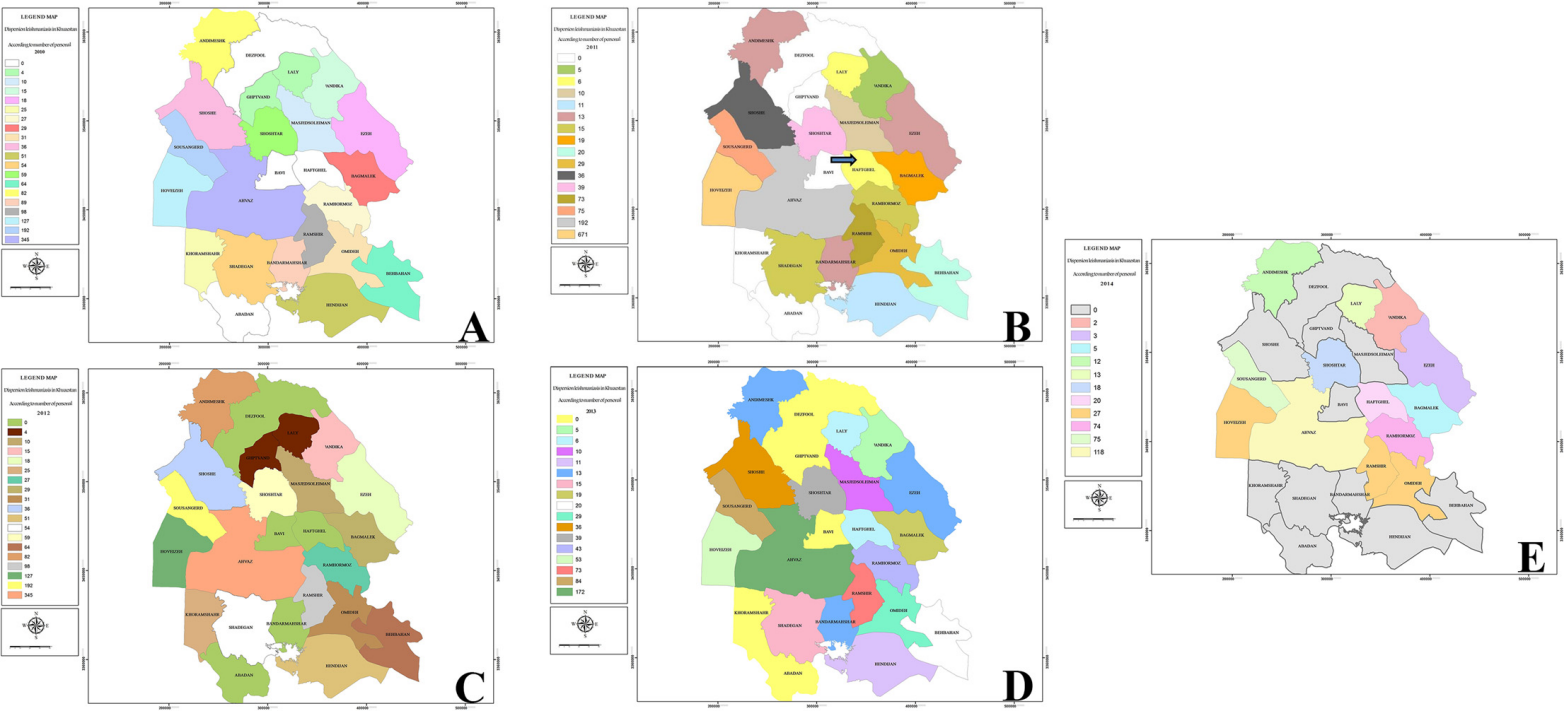
(A) Incidence of cutaneous leishmaniasis (CL) using geographical information system in (GIS) 2010, (B) GIS showed a new focus of CL in 2011 compared to 2010 (arrow), (C) The highest incidence of CLin 2012 with 1363 patients of CL has shown in Khuzestan Province with GIS mapping, (D) Geographical distribution mapping of CL incidence in 2013, E: The incidence rate of CL was diminished after control programs. Ten distinct had no reported of the disease in 2014.

**Fig 2:**
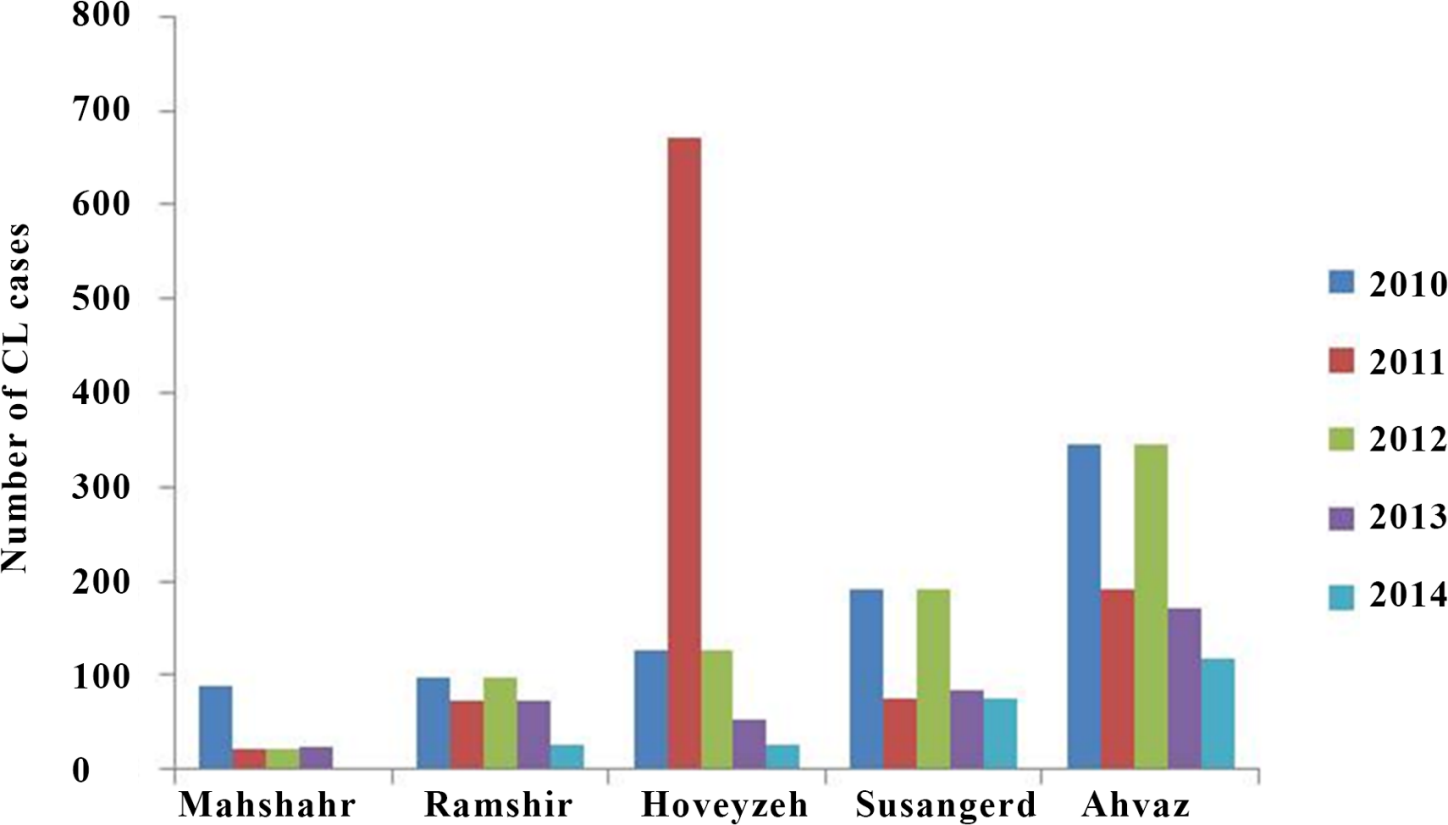
CL incidence in the five districts with most cases from 2010 to 2014. At the latest census, population of the cities is 1,112,021; 109,927; 43,591; 14,422 for Ahvaz, Mashshahr, Susangerd, Hoveyzeh and 24,782, respectively.

The collected data showed that majority of the intensive events in the five years of the study was belonged to the Ahvaz followed by Hoveizeh and Sousangerd where are located in center and eastern districts of Khuzestan. Data of GIS referring to CL patients showed that these three cities had a significant number of cases (2010: 664 cases (57%) of the population had involved with CL, 2011: 938 (88%), 2012: 664 (48.7%), 2013: 329 (49.8%). Amongst the five years period of the study, year 2012 with 1363 patients (29.1%) and 2014 with 420 patients (8.98%) showed the highest and lowest number of cases, respectively (Table 1). Distribution maps of CL in Khuzestan Province by the number of patients in during the study period are presented in Fig 1A–1E. Overall CL incidence that was recorded by CDC in the five districts with most cases from 2010 to 2014 is shown in Fig 2.

**Table 1:** The incidence of cutaneous leishmaniasis in Khuzestan Province during the period study.

Month/year	2010	2011	2012	2013	2014	Total
January	241	276	283	65	113	978
February	190	162	207	66	56	681
March	85	70	65	97	24	341
April	55	35	41	51	19	201
May	44	25	35	49	15	168
June	25	23	27	27	8	110
July	60	54	47	22	13	196
August	71	52	63	36	15	207
September	36	44	76	65	16	237
October	104	89	101	61	13	368
November	110	117	138	47	43	455
December	145	115	280	75	85	700
Total	1166	1062	1363	661	420	4672

There was no report of the disease in Dezul during of this study. Based on GIS there was a new focus of CL in 2011 compared to 2010 (Fig 1A). In 2014 that control program was done, ten distinct of Khuzestan Province didn’t show any cases of the disease (Fig 1E). Seasonality of CL is mainly restricted to October–February period with the main peaks occurring in December and January (Table 1). In general, in the period of this study, the highest number of cases were observed in January (20.93%), December (14.98.1%) and February (14.57%), November (9.73%), and October (7.87%) respectively.

## Discussion

The primarily aim of this study was to produce distributional maps of CL over five years and evaluate the role of GIS in control of CL in Khuzestan province where an endemic area of CL in Iran is. Cutaneous leishmanisis is found worldwide and is considered to be endemic in 88 countries [18] such as Iran [2]. It has been reported in 20 of the 31 provinces of Iran [5]. According to the classification of World Health Organization (WHO) leishmaniasis is neglected disease of great epidemiological importance, which requires effective control measures. They present potential to epidemic outbreaks because of their transmission by vector insects [19]. Here, we presented the distribution of CL in Khuzestan Province at a period of 4 years, 2010–2013, and compared its distribution with 2014 in which control programs of CL was done based on GIS information. Leishmaniasis is hyper endemic in the southwestern region of Iran, in which Khuzestan Province is located [20]. A development of the GIS has provided powerful tools to investigate distribution patterns in studying infectious diseases such as leishmaniasis [21]. This is the first study to investigate distribution pattern and the possibility control of CL by using GIS.

This study reports a heterogeneous distribution and fluctuating trend of CL incidence with a higher incidence in Ahvaz, Hoveizeh and Sousangerd where are located in center and eastern districts of Khuzestan. Data of GIS referring to CL patients showed that these three cities had a significant number of cases (2010: 664; 2011: 938; 2012: 664; 2013: 329 cases). Several studies predict an increasing trend of the epidemic potential and the transmission season of vector born disease in temperate regions due to climate change and changes activities of reservoirs and vectors [22–24]. Climate variability may have different impact in the transmission of CL depending on the various *Leishmania* species and the particular vectors in different regions of the world [25]. Climatic factors (e.g. temperature, rainfall and vegetation cover) and human interventions (e.g. deforestation, building of dams and urbanization) are the most known effecting environmental factors on CL occurrence [19, 26]. In addition, leishmaniasis outbreak is related to human activities close to or within forested areas: irrigation schemes and horticulture development, building of dams, road construction, and establishment of new residential colonies lead to intrusion into the sylvatic cycle of the disease. In addition, leishmaniasis outbreak is related to human activities close to or within forested areas: road construction, building of dams, irrigation schemes and horticulture development, and establishment of new residential colonies lead to intrusion into the sylvatic cycle of the disease [26]. In this study, the endemic areas of CL from during 2010–2013 were recognized using GIS maps and the control programs done in these regions. In 2014, the incidence and geographical distribution of CL was investigated to consider success of control programs associated with GIS. We found that the mean incidence of CL was 1063 (2010–2013) and 420 (2014) cases in pre and post implementation of control programs, respectively. There were also no cases with the disease in ten distinct of Khuzestan Province in 2014 (Fig 1D) in which the control programs had been done. Additionally, the incidence rate of the disease in three cities with high occurrence was diminished from mean 649 cases annually over four years, 2010–2013, to 267 cases in 2014 after implementation of control programs. In the other words, amongst the five years period of the study, year 2012 with 1363 patients and 2014 with 420 patients showed the highest and lowest number of cases, respectively. In 2011, we found new focus of CL compared to 2010. This may explain with climate variability. Modeling studies in southwest Asia have indicated potential range expansion of *Phlebotomus papatasi* with global warming [27]. Low temperature appears to be one of the main factors to its spread to new regions [28]. The data obtained from seasonality incidence of CL showed the highest number of cases in January and December as well as previous reported [2, 7].

In conclusion, analyses of data distributed in the geographic spaces are increasingly appreciated in leishmaniasis control management. Descriptive analyses of control measures allowed to evaluating that the information system and GIS tools promoted greater efficiency in making decisions and planning activities in the control of vector born disease such as leishmaniasis. This tool also provided appreciated information to the necessity of new approaches to the control of CL in the endemic areas.
